# Editorial: Advances in cAMP signaling research: basic and translational aspects

**DOI:** 10.3389/fphys.2023.1266718

**Published:** 2023-09-01

**Authors:** Muhammad Aslam, Yury Ladilov

**Affiliations:** ^1^ Experimental Cardiology, Department of Internal Medicine I, Justus Liebig University, Giessen, Germany; ^2^ Department of Cardiology, Kerckhoff Clinic GmbH, Bad Nauheim, Germany; ^3^ DZHK (German Centre for Cardiovascular Research), Partner Site Rhein-Main, Bad Nauheim, Germany; ^4^ Department of Cardiovascular Surgery, Heart Center Brandenburg, Brandenburg Medical School Theodor Fontane, Bernau bei Berlin, Germany

**Keywords:** soluble adenylyl cyclase, compartmentalization, PDEs, PKA, EPAC, PKD, polycystic kidney disease, ciliopathy

Even though 3′–5′-cyclic adenosine monophosphate (cAMP) was discovered over half a century ago, it continues to be an object of scientific interest. This is not surprising, since cAMP signaling plays an important role in a wide variety of physiological processes, such as transcription regulation, metabolism, mitochondrial homeostasis, as well as cell growth and cell death. The importance of cAMP signaling is further highlighted by its evolutionary conservation, spanning from microorganisms to mammals (Gancedo, 2013).

Over the last decade, research on cAMP signaling has unveiled a highly organized compartmentalization of cAMP within the cell, contributing to the specificity and selectivity to its signaling (Zaccolo et al., 2021). This compartmentalization occurs at multiple levels (Aslam and Ladilov, 2022): Firstly, the distinct spatial distribution of adenylyl cyclases (ACs) plays a role. The membrane-localized AC (tmAC) predominately generates cAMP in close proximity to the plasma membrane (Halls and Cooper, 2017), while soluble AC (sAC), localizes within various cellular organelles forming cAMP pools in various cellular compartments, such as cytosol, mitochondria, and nucleus (Zippin et al., 2003; Appukuttan et al., 2012; Watson et al., 2015). Secondly, cAMP hydrolyzing phosphodiesterases (PDEs) contribute to the compartmentalization of cAMP signaling by being localized near sites of cAMP generation, limiting its diffusion to other compartments (Mika et al., 2012; Zhang et al., 2019). Lastly, scaffolding proteins such as A-kinase anchoring proteins (AKAPs) play a role in connecting cAMP synthesis to functional effectors like protein kinase A (PKA) and exchange protein directly activated by cAMP (Epac). AKAPs exhibit the ability to bind not only PKA but also other kinases, cyclases, PDEs, G-protein-coupled receptors, and phosphatases (Zaccolo et al., 2021). Consequently, AKAPs, localized at specific subcellular sites, play a crucial role in maintaining the subcellular compartmentalization of cAMP signaling.

In this context, a study by Cattani-Cavalieri et al. published in this Research Topic focuses on understanding the signaling networks in Human Airway Smooth Muscle (HASM) cells, particularly those related to cAMP signaling pools coming from two distinct AC isoforms, i.e., AC2 and AC6. The cAMP signaling in HASM has enormous translational significance, as it involves in bronchodilation by stimulation of β2-adrenoreceptors, e.g., in patients with bronchial asthma and chronic obstructive pulmonary disease. Although G-protein coupled receptors (GPCR) expressed in HASM cells utilize cAMP signaling, activation of different receptors leads to responses that are distinct from those elicited by β2-adrenoreceptors. This phenomenon may be due to the compartmentalization of cAMP signaling within the cell. To investigate this issue, the authors applied Stable Isotope Labeling of Amino acids in Cell culture (SILAC) technique to quantify phosphorylated proteins in HASM. Stimulating the AC activity, the authors found that 14 differentially phosphorylated proteins (DPPs) resulted from AC2 activity and 34 differentially phosphorylated proteins resulted from AC6 activity. They also revealed that AC2 signaling is associated with modifications in RNA/DNA binding proteins and microtubule/spindle body proteins, whereas AC6 signaling is associated with proteins regulating autophagy, calcium-calmodulin signaling, Rho GTPases, and cytoskeletal regulation. Noteworthy, the phosphorylation of OFD1 exhibited contrasting regulation, showing increased phosphorylation at Ser899 in response to AC6 activation, while AC2 activation resulted in decreased phosphorylation of the same site. The study thus provides new insight into the compartmentalization of cAMP signaling within the cell.

In another study, Paolocci and Zaccolo delve into the Research Topic of compartmentalized cAMP signaling within the primary cilium and explore the utilization of optogenetic tools to gain insights into the mechanisms underlying ciliopathy in context of autosomal dominant polycystic kidney disease (PKD). The cilium serves as a hub for cell signaling, housing various pathways, including cAMP signaling. Any disruption of cAMP signaling within the cilium can lead to perturbed cytogenesis, potentially resulting in pathological conditions like PKD. Notably, several GPCRs, such as Gpr161 and Gpr175, act as AKAPs facilitating the compartmentalization of cAMP signaling at the primary cilium. Paolocci and Zaccolo discuss various methodologies developed and employed to investigate compartmentalized cAMP signaling in the primary cilium. Among these tools, two FRET-based reporters have been considered: the Epac-based reporter, which measures cAMP production (Klarenbeek et al., 2015) and the AKAP-based reporter, which measures localized PKA activity (Depry and Zhang, 2011). Additionally, to achieve spatiotemporal activation of cAMP signaling in specific experiments, researchers employed site-specific ectopic expression of photoactivatable ACs (Stierl et al., 2011).

Various components of the cAMP signaling are important targets for translational approach. Particularly, the inhibition of sAC may be a promising approach in several therapeutic applications (Ladilov and Appukuttan, 2014; Aslam and Ladilov, 2020). Furthermore, the significance of the sAC inhibitors as on-demand, non-hormonal male contraceptives has also been recently considered (Ferreira et al., 2022). To develop the sAC-specific inhibitor a reliable assay is required. In the Research Topic, Rossetti et al. described sAC-focused *in vitro* assays to identify and characterize sAC inhibitors for therapeutic use. The assays include tests for various parameters, like potency, binding kinetics, and residence times of the inhibitors, as well as a cellular potency assay to test the functional effects of the inhibitors in a cellular model. Thus, the authors described the workflow designed to identify sAC inhibitors with improved therapeutic properties, such as high potency and long residence times, and thereby develop drugs for the therapeutic application of sAC inhibition.

Lastly, Zhang et al. provide a comprehensive overview about the role and mechanisms of perturbed cAMP signaling in neurodegenerative disorders such as Parkinson’s disease (PD) and Alzheimer’s disease (AD). In neurodegenerative disorders, excessive production of reactive oxygen species (ROS) is regarded as one of the major mechanisms leading to neuronal cell death (Quan et al., 2020). The authors discuss and elaborate on the role of ROS in various forms of regulated cell death (RCD) such as parathanatos, NETosis, ferroptosis and lysosome-dependent cell death. Cellular cAMP signaling exerts differential effects on various forms of RCD e.g., cAMP/PKA signaling inhibits NETosis and cell senescence while cAMP/PKA and Epac promote parthanatos and induce lysosomal cell death. In AD and PD, cAMP signaling is perturbed resulting in the facilitation of one or more of these ROS-mediated RCD processes. Understanding the distinct roles of cAMP signaling and its effectors in different cellular compartments will be crucial for developing effective therapies for neurodegenerative diseases like AD and PD. The study provides detailed and up-to-date knowledge about the role of cAMP signaling in RCD and discusses potential therapeutic targets.

In summary, this Research Topic highlights some actual trends in both basic and translational research concerning cAMP signaling. This holds particular significance in the context of diseases such as bronchial asthma, chronic obstructive pulmonary disease and PKD, where the compartmentalization of cAMP signaling plays a crucial role. Moreover, the Research Topic highlights the impact of impaired cAMP signaling in various forms of cell death, ultimately contributing to the development of neurodegenerative diseases. Lastly, the Research Topic presents several methodological approaches, including the utilization of FRET-based reporters to study cAMP compartmentalization, and the identification and characterization of sAC inhibitors for potential therapeutic applications through *in vitro* assays.

## References

[B1] Appukuttan A. Kasseckert S. A. Micoogullari M. Flacke J. P. Kumar S. Woste A. (2012). Type 10 adenylyl cyclase mediates mitochondrial Bax translocation and apoptosis of adult rat cardiomyocytes under simulated ischaemia/reperfusion. Cardiovasc Res. 93, 340–349. 10.1093/cvr/cvr306 22106416

[B2] Aslam M. Ladilov Y. (2022). Emerging role of cAMP/AMPK signaling. Cells 11, 308. 10.3390/cells11020308 35053423 PMC8774420

[B3] Aslam M. Ladilov Y. (2020). Targeting the sAC-dependent cAMP pool to prevent SARS-cov-2 infection. Cells 9, 1962. 10.3390/cells9091962 32854430 PMC7563949

[B4] Depry C. Zhang J. (2011). Using FRET-based reporters to visualize subcellular dynamics of protein kinase A activity. Methods Mol. Biol. 756, 285–294. 10.1007/978-1-61779-160-4_16 21870233 PMC4386889

[B5] Ferreira J. Levin L. R. Buck J. (2022). Strategies to safely target widely expressed soluble adenylyl cyclase for contraception. Front. Pharmacol. 13, 953903. 10.3389/fphar.2022.953903 36091839 PMC9452739

[B6] Gancedo J. M. (2013). Biological roles of cAMP: variations on a theme in the different kingdoms of life. Biol. Rev. Camb Philos. Soc. 88, 645–668. 10.1111/brv.12020 23356492

[B7] Halls M. L. Cooper D. M. F. (2017). Adenylyl cyclase signalling complexes - pharmacological challenges and opportunities. Pharmacol. Ther. 172, 171–180. 10.1016/j.pharmthera.2017.01.001 28132906

[B8] Klarenbeek J. Goedhart J. van Batenburg A. Groenewald D. Jalink K. (2015). Fourth-generation epac-based FRET sensors for cAMP feature exceptional brightness, photostability and dynamic range: characterization of dedicated sensors for FLIM, for ratiometry and with high affinity. PloS one 10, e0122513. 10.1371/journal.pone.0122513 25875503 PMC4397040

[B9] Ladilov Y. Appukuttan A. (2014). Role of soluble adenylyl cyclase in cell death and growth. Biochimica biophysica acta 1842, 2646–2655. 10.1016/j.bbadis.2014.06.034 25010002

[B10] Mika D. Leroy J. Vandecasteele G. Fischmeister R. (2012). PDEs create local domains of cAMP signaling. J. Mol. Cell. Cardiol. 52, 323–329. 10.1016/j.yjmcc.2011.08.016 21888909

[B11] Quan H. Koltai E. Suzuki K. Aguiar A. S. Jr. Pinho R. Boldogh I. (2020). Exercise, redox system and neurodegenerative diseases. Biochim. Biophys. Acta Mol. Basis Dis. 1866, 165778. 10.1016/j.bbadis.2020.165778 32222542

[B12] Stierl M. Stumpf P. Udwari D. Gueta R. Hagedorn R. Losi A. (2011). Light modulation of cellular cAMP by a small bacterial photoactivated adenylyl cyclase, bPAC, of the soil bacterium Beggiatoa. J. Biol. Chem. 286, 1181–1188. 10.1074/jbc.M110.185496 21030594 PMC3020725

[B13] Watson R. L. Buck J. Levin L. R. Winger R. C. Wang J. Arase H. (2015). Endothelial CD99 signals through soluble adenylyl cyclase and PKA to regulate leukocyte transendothelial migration. J. Exp. Med. 212, 1021–1041. 10.1084/jem.20150354 26101266 PMC4493416

[B14] Zaccolo M. Zerio A. Lobo M. J. (2021). Subcellular organization of the cAMP signaling pathway. Pharmacol. Rev. 73, 278–309. 10.1124/pharmrev.120.000086 33334857 PMC7770493

[B15] Zhang L. Bouadjel K. Manoury B. Vandecasteele G. Fischmeister R. Leblais V. (2019). Cyclic nucleotide signalling compartmentation by PDEs in cultured vascular smooth muscle cells. Br. J. Pharmacol. 176, 1780–1792. 10.1111/bph.14651 30825186 PMC6514293

[B16] Zippin J. H. Chen Y. Nahirney P. Kamenetsky M. Wuttke M. S. Fischman D. A. (2003). Compartmentalization of bicarbonate-sensitive adenylyl cyclase in distinct signaling microdomains. FASEB J. 17, 82–84. 10.1096/fj.02-0598fje 12475901

